# Ventricular tachycardia and acute heart failure induced by atropine in the treatment of bradycardia: A case report and literature review

**DOI:** 10.1097/MD.0000000000034775

**Published:** 2023-08-25

**Authors:** Huanhuan Zhang, Meng Zhang, Yanru Du, Jinhua He, Jianli Li

**Affiliations:** a Department of Anesthesiology, Hebei General Hospital, Shijiazhuang City, Hebei Province, China.

**Keywords:** acute heart failure, atropine, bradycardia, heart rate variability, ventricular tachycardia

## Abstract

**Rationale:**

Despite various advantages of laparoscopic surgical procedures, artificial pneumoperitoneum might lead to hemodynamic fluctuations including severe bradycardia and cardiac arrest. Atropine is usually proposed to treat intraoperative severe bradycardia ( < 40 beats per minute). However, atropine could induce ventricular arrhythmias, which might be life-threatening in severe case.

**Patient concerns:**

Here, we reported a 41-year-old female who was diagnosed with gallbladder polyps and was scheduled for laparoscopic cholecystectomy under general anesthesia.

**Diagnoses:**

Bradycardia occurred suddenly during the operation and atropine was injected intravenously. Eventually the patient developed ventricular tachycardia and acute heart failure.

**Interventions:**

We organized an urgent consultation and the patient was treated immediately.

**Outcomes:**

Fortunately, the patient experienced no complications after timely diagnosis and treatment. After 6 months of follow-up, her New York Heart Association classification was I with no complications.

**Lessons:**

This case highlighted that the administration of atropine to treat bradycardia may lead to ventricular tachycardia and acute heart failure, and anesthesiologists should remain vigilant to avoid potentially life-threatening consequences.

## 1. Introduction

Laparoscopic surgery has been widely used for cholecystectomy due to its advantages of minimally invasive, mild pain, and short hospital duration.^[1]^ However, artificial abdominal gas insufflation could result in bradycardia or even cardiac arrest, which was associated with an increase in vagus nerve activity arised from peritoneal stretch.^[2]^ Bradycardia is defined as heart rate (HR) of < 60 beats per minute (bpm).^[3]^ Atropine, as an anticholinergic drug, was recommended for the treatment of bradycardia for decades.^[4]^ However, no studies reported ventricular arrhythmia and acute heart failure induced by atropine in the treatment of bradycardia during laparoscopic cholecystectomy under general anesthesia.

Here, we reported a rare case of acute heart failure following ventricular tachycardia after atropine therapy for bradycardia during artificial abdominal gas insufflation. And we performed a case review of ventricular arrhythmia and acute heart failure with the treatment of atropine. This case report conforms to CARE guidelines.^[5]^

## 2. Case presentation

**Figure 1.**
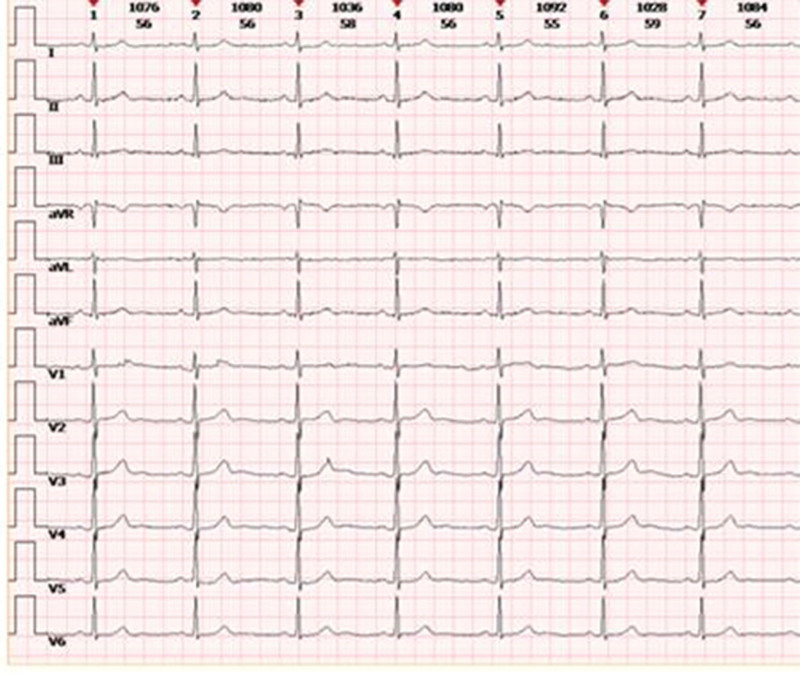
The preoperative 12-lead electrocardiogram showed sinus rhythm with a rate of 56 bpm, PR interval 162 ms, and Q-T interval 422 ms. bpm = beats per minute.

A 41-year-old female (83 kg, 168 cm, American Society of Anaesthesiologists status II) with a 1-year history of gallbladder polyps was scheduled for laparoscopic cholecystectomy. She had the history of anemia and received ferrous succinate tablets (0.1 g, bid) treatment. The anemia was cured on her admission (hemoglobin 134 g/L) and ferrous succinate tablets were stopped. The blood pressure was controlled around 130/80 mm Hg. She had no food or medication allergies, and denied alcohol or tobacco consumption. The preoperative electrocardiogram (ECG) showed sinus bradycardia (HR of 56 bpm), PR interval 162 ms, and Q-T interval (QTc) 422 ms. (Fig. 1) Computed tomography scan of the abdomen showed multiple gallbladder polyps. The preoperative chest computed tomography, echocardiography, and laboratory tests showed no obvious abnormalities.

After the patient entering the operating room, routine monitors were initiated, including 5-lead ECG, noninvasive blood pressure, SPO_2_, and bispectral index. Before anesthesia, vital signs presented as HR of 70 to 80 bpm and blood pressure of 130/78 mm Hg. Anesthesia induction was achieved by midazolam (2 mg), sufentanil (25 ug), cisatracurium besylate (16 mg), and etomidate (16 mg). After intubation, anesthesia was maintained with remifentanil (0.05–0.15 µg/kg/minutes), propofol (2–4 mg/kg/hours), combined with 1% sevoflurane, keeping the bispectral index between 40 and 60.

**Figure 2.**
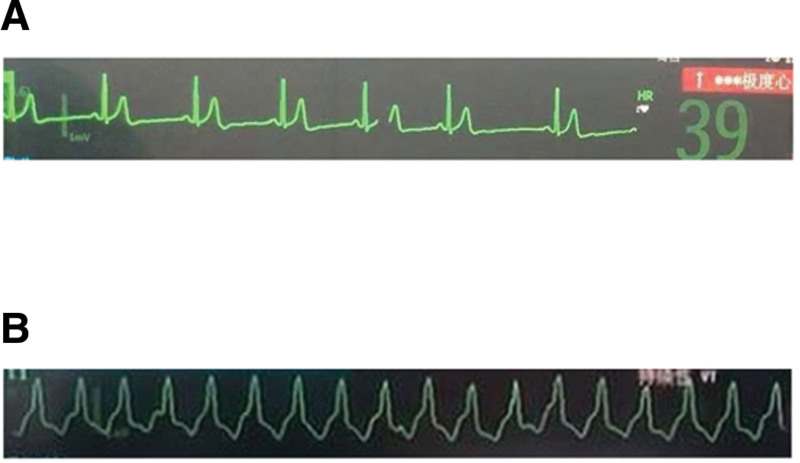
(A) Electrocardiogram of bradycardia at the moment of pneumoperitoneum was reestablished after the gallbladder was removed. (B) Electrocardiogram of ventricular tachycardia happened after the use of atropine.

From the induction of anesthesia to the removal of the gallbladder, the vital signs were relatively stable, HR fluctuated between 60 to 80 bpm and blood pressure ranged from 104/55 to 135/80 mm Hg. However, at the moment of artificial abdominal gas insufflation was reestablished after the gallbladder was removed, HR suddenly dropped to 39 bpm (Fig. 2A), and atropine 0.5 mg was injected intravenously. Unfortunately, there was a significant increase in HR (HR from 39–160 bpm) and premature ventricular contraction occurred in the monitor, ultimately developing ventricular tachycardia (Fig. 2B). The patient was treated with esmolol 20 mg twice intravenously. When the hemodynamic became relatively stable (HR: 100 bpm, blood pressure: 130/85 mm Hg), the operation was continued and successfully completed. After the operation, the tracheal tube was removed and the patient was transferred to the postanesthesia care unit.

**Figure 3.**
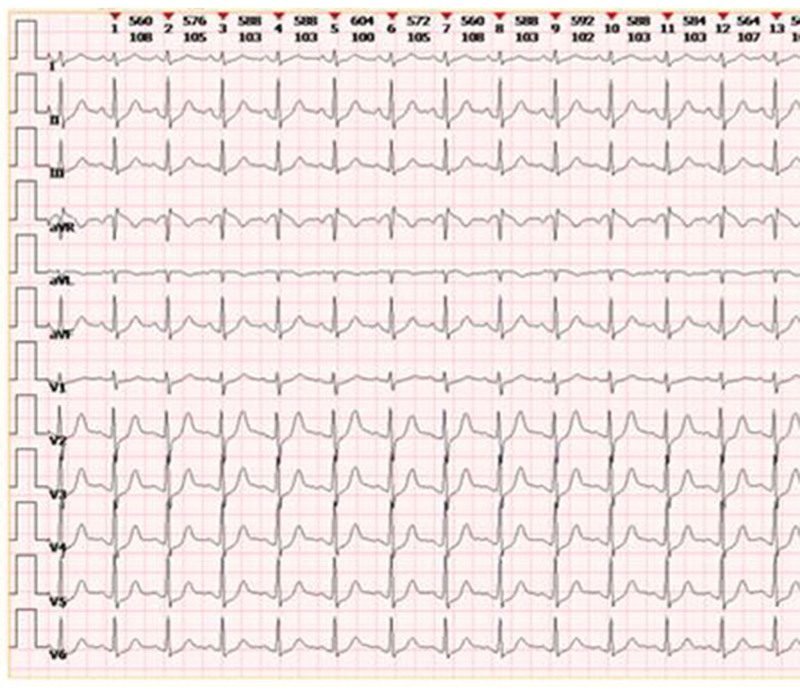
The postoperative 12-lead electrocardiogram showed sinus tachycardia with a rate of 103 bpm and ST-T segment depression in the postanesthesia care unit. bpm = beats per minute.

After entering the postanesthesia care unit, HR fluctuated between 90 to 100 bpm, but blood pressure decreased from 110/73 to 95/60 mm Hg gradually and the patient showed slight shortness of breath. Blood pressure increased slightly after phenylephrine 100 µg was used intravenously, but soon dropped to 90/60 mm Hg. We organized an urgent consultation with the cardiologist and the sonographer. The postoperative 12-lead ECG showed sinus tachycardia with a rate of 103 bpm and ST-T segment depression. (Fig. 3) Bedside echocardiography showed abnormal, with reduced left ventricular anterior and inferior wall motion, reduced left ventricular systolic function, left ventricular dilatation, left ventricular diastolic dysfunction, and an ejection fraction of 38%. Immediately, the patient received a continuous intravenous pump of dopamine (8–10µg/kg/minutes) and was transferred to the intensive care unit for further treatment.

In the intensive care unit, the myocardial enzymes, pro-brain natriuretic peptide, and high-sensitivity troponin T in serum were measured instantly. The levels of pro-brain natriuretic peptide and high-sensitivity troponin T were 3250 ng/L (normal range < 450 ng/L) and 0.627 ng/mL (normal range < 0.1 ng/mL), then subsequently returning back to normal within 4 days. Creatine kinase and creatine kinase-MB peaked at 237.0 units/L (normal range 40–200 units/L) and 58.5 units/L (normal range 0–19 units/L), returning back to normal within 2 days. Eventually, acute heart failure was diagnosed according to the 2021 European Society of Cardiology Heart Failure Guidelines.^[6]^ Fortunately, after the administration of dopamine and recombinant human brain natriuretic peptide, the patient’s cardiac function was improved and echocardiography showed no obvious abnormalities with ejection fraction recovering to 65% on the 4th day after surgery.

The patient was discharged 4 days later and experienced no further symptoms. After 6 months of follow-up, her New York Heart Association classification was I with no complications.

## 3. Discussion

Laparoscopic surgery was considered as an increasingly common surgical procedure with less postoperative pain and faster recovery.^[7]^ However, extensive physiological changes induced by pneumoperitoneum, such as increased intra-abdominal pressure, carbon dioxide accumulation, and dramatic hemodynamic fluctuations, should be appreciated by anesthesiologists.^[8]^ Studies reported that peritoneal insufflation accompanied with rapid peritoneum stretch could provoke a vagal-mediated cardiovascular reflex, which might lead to bradycardia or even asystole.^[2,9]^ Bradycardia was considered as an early warning sign for cardiac arrest during routine laparoscopic surgery.^[4]^ Therefore, it is extremely important for anesthesiologists to manage bradycardia in laparoscopic surgeries under general anesthesia to prevent devastating consequences.

It was reported that sudden bradycardia was often caused by an abrupt activation of vagal reflex, and it could be treated by atropine administration.^[10]^ Atropine was proposed to treat bradycardia by blocking cardiac parasympathetic nerve.^[11,12]^ In our case, we observed a sudden drop in HR from 60 bpm to 39 bpm and injected atropine 0.5 mg instantly. Unfortunately, HR increased significantly and premature ventricular contraction occurred in the monitor, ultimately developing ventricular tachycardia (HR: 160 bpm). Previous reports presented 3 cases of tachyarrhythmias induced by atropine,^[13–15]^ and the results were shown in Table 1. However, none of these case reports occurred during laparoscopic surgery. Although there were numerous reports on tachyarrhythmias after the administration of atropine, the precise mechanism remains ambiguous.

**Table 1:** Previous cases of ventricular arrhythmia induced by atropine in the treatment of bradycardia.

First author	HR before atropine treatment	Type of arrhythmia	The dosage of atropine	Operation procedure
Lunde^[13]^	Between 30 and 35 bpm	Ventricular fibrillation	0.5 mg	-
Cooper^[14]^	Between 45 and 55 bpm	Ventricular fibrillation	0.5 mg	-
Lee^[15]^	Between 42 and 45 bpm	Ventricular tachycardia	0.5 mg	A varicosectomy

bpm = beats per minute, HR = heart rate.

Several studies demonstrated that he imbalance of autonomic nervous system was responsible for ventricular tachyarrhythmias after atropine therapy.^[16,17]^ In recent decades, heart rate variability analysis was widely used to evaluate the sympathovagal balance.^[18]^ In a previous case report we presented, heart rate variability analysis showed that there was an increase in sympathetic activity and a decrease in parasympathetic activity after atropine was injected.^[19]^ Another case report indicated that atropine-related ventricular tachycardia might be attributed to a decrease in parasympathetic activity and then elite unopposed sympathetic activity.^[16]^ The above evidence indicated that increased sympathetic activity might contribute to ventricular tachycardia in our case.

Additionally, as the studies reported, patients with significant ischemic heart disease were prone to ventricular arrhythmias after atropine treatment.^[17,20]^ The case we presented was absent of ischemic heart disease before the operation, which made it reasonable to use atropine. However, ventricular tachycardia induced by atropine increased myocardial oxygen consumption, shortened the diastole, and decreased coronary perfusion, which might induce myocardium damage leading to acute heart failure.^[21]^ Acute heart failure was a threaten to the patient and required immediate treatment.^[22]^

It was well known to us that bradycardia was an undesirable event and intraoperative HR below 50 bpm required treatment. However, ventricular tachycardia and acute heart failure occurred after the treatment of bradycardia using atropine. In this case, the patient’s symptoms, bedside echocardiography, postoperative ECG and laboratory indexes all supported the diagnosis of acute heart failure. Fortunately, we organized multidisciplinary consultations promptly after surgery, which ensured that the patient received timely diagnosis and treatment without complications.

The limitation of this case report was that only atropine was used to treat bradycardia. Randomized controlled studies are needed in the future to determine which drug is more suitable for treating bradycardia during laparoscopic cholecystectomy. Regarding the potentially catastrophic consequences of atropine administration, we think that our case had educational value to make many anesthesiologists remain vigilant when using atropine for bradycardia during laparoscopic cholecystectomy.

## 4. Conclusion

As this case presented, the patient developed ventricular tachycardia and acute heart failure after atropine therapy for bradycardia during laparoscopic cholecystectomy. It was critical for anesthesiologists to monitor vital signs closely during the administration of atropine in case of devastating outcomes.

## Acknowledgements

The authors thank this patient for providing this medical information.

## Author contributions

**Conceptualization:** Jianli Li.

**Data curation:** Huanhuan Zhang, Meng Zhang.

**Investigation:** Huanhuan Zhang, Yanru Du.

**Writing – original draft:** Huanhuan Zhang.

**Writing – review & editing:** Meng Zhang, Jinhua He, Jianli Li.
